# Susceptibility to COVID-19 Diagnosis in People with Down Syndrome Compared to the General Population: Matched-Cohort Study Using Primary Care Electronic Records in the UK

**DOI:** 10.1007/s11606-022-07420-9

**Published:** 2022-04-06

**Authors:** R. Asaad Baksh, Andre Strydom, Sarah E. Pape, Li F. Chan, Martin C. Gulliford

**Affiliations:** 1 Department of Forensic and Neurodevelopmental Sciences, Institute of Psychiatry, Psychology, and Neuroscience, King’s College London, London, UK; 2 South London and Maudsley NHS Foundation Trust, London, UK; 3 Present Address: The LonDowns Consortium, London, UK; 4 Centre for Endocrinology, William Harvey Research Institute, Barts and the London School of Medicine, Queen Mary University of London, Charterhouse Square, London, UK; 5 School of Population Health and Environmental Sciences, King’s College London, London, UK

## Abstract

**Background:**

During the COVID-19 pandemic, people with Down syndrome (DS) have experienced a more severe disease course and higher mortality rates than the general population. It is not yet known whether people with DS are more susceptible to being diagnosed with COVID-19.

**Objective:**

To explore whether DS is associated with increased susceptibility to COVID-19.

**Design:**

Matched-cohort study design using anonymised primary care electronic health records from the May 2021 release of Clinical Practice Research Datalink (CPRD) Aurum.

**Setting:**

Electronic health records from approximately 1400 general practices (GPs) in England.

**Participants:**

8854 people with DS and 34,724 controls matched for age, gender and GP who were registered on or after the 29th January 2020.

**Measurements:**

The primary outcome was COVID-19 diagnosis between January 2020 and May 2021. Conditional logistic regression models were fitted to estimate associations between DS and COVID-19 diagnosis, adjusting for comorbidities.

**Results:**

Compared to controls, people with DS were more likely to be diagnosed with COVID-19 (7.4% vs 5.6%, *p* ≤ 0.001, odds ratio (OR) = 1.35; 95% CI = 1.23–1.48). There was a significant interaction between people with DS and a chronic respiratory disease diagnosis excluding asthma and increased odds of a COVID-19 diagnosis (OR = 1.71; 95% CI = 1.20–2.43), whilst adjusting for a number of comorbidities.

**Conclusion:**

Individuals with DS are at increased risk for contracting COVID-19. Those with underlying lung conditions are particularly vulnerable during viral pandemics and should be prioritised for vaccinations.

## INTRODUCTION

The COVID-19 pandemic caused by SARS-CoV-2 infection has disproportionately affected people with intellectual disabilities with increased hospital admissions for severe disease and mortality.^1^ Down syndrome (DS), caused by trisomy of part or all of chromosome 21, is the most common genetic cause for intellectual disability, with a worldwide prevalence of approximately 1:1000.^2^ Individuals with DS often present with a range of comorbidities that may increase the risk for developing more severe COVID-19 infection, including congenital heart conditions, a higher prevalence of obesity, respiratory tract infections and sleep apnoea,^3,4^ and Alzheimer’s disease.^5^ In addition, it has been proposed that elevated levels of the proinflammatory cytokines IL-6 and TNF-α, and enhanced interferon signalling associated with IFNAR1, IFNAR2 and IFNGR2 overexpression, may increase risk for severe outcomes for COVID-19 by increasing the likelihood of an abnormal cytokine response.^6^

Some triplicated genes and their knock-on effects in individuals with DS may increase the susceptibility for viral infection, including for SARS-CoV-2. Triplication of *TMPRSS2*, located on chromosome 21, may enhance viral entry in DS and thus increase the likelihood of infection with SARS-CoV-2 as it interacts with the viral S protein to allow SARS-CoV-2 entry into the host cells.^7^ Viral diseases that use ACE-2-receptor/TMPRSS2 for cell binding/cell entry include influenza, SARS-CoV-1, metapneumovirus and MERS. Social and behavioural factors, including lower adherence to social distancing and handwashing recommendations, might also contribute to SARS-CoV-2 transmission in DS. Many individuals with DS, and particularly older adults, may be at higher risk for infection due to increased exposure through living in care settings and through contact with rotating care teams.^8^

Research shows that people with DS are more likely to have severe COVID-19 outcomes, with hospitalisation rates approximately 5 times higher than those of the general population^9^. Mortality rates are approximately 3 times higher than those of controls even after adjusting for known risk factors for COVID-19 mortality.^10^ Furthermore, COVID-19 patients with DS present with significantly higher rates of medical complications, particularly lung complications such as pneumonia and acute respiratory distress syndrome.^10^

Whether DS is associated with increased susceptibility to SARS-CoV-2 infection is unknown but could have important implications for prevention of infection given the increased risk for poor outcomes. We aimed to establish if individuals with DS of all ages were more likely to be diagnosed with COVID-19 compared to age- and gender-matched peers in a population-based cohort study drawing on primary care electronic health records from the UK from January 2020 to May 2021. We aimed to identify comorbidities associated with risk, as well as to explore the potential role of other associated factors such as living situation.

## METHODS

### Study Population and Data Sources

We conducted a cohort study using anonymised primary care electronic health records from the May 2021 release of Clinical Practice Research Datalink (CPRD) Aurum, which is a population-based database of the electronic health records for approximately 1400 general practices (GPs) in England. CPRD Aurum was made available for research purposes in 2018 and, as of March 2021, consists of data from over 39 million patients with over 13 million currently registered. The database contains data from GPs in England using the EMIS clinical systems and includes data on diagnoses, symptoms, prescriptions, referrals and tests.^11,12^ Previous studies have confirmed the quality of data from CPRD Aurum.^13–15^ This study protocol was approved by the CPRD Independent Scientific Advisory Committee (ISAC application number 20_099).

We utilised a matched-cohort study design.^16^ Patients with DS ever recorded were eligible if they were registered at a GP on or after the 29th of January 2020, the date of the UK’s first confirmed COVID-19 case. DS patients aged 80 and over were excluded as this exceeds the typical life expectancy of individuals with DS.^17,18^ Up to four control participants without DS were sampled with replacement from the list of all patients registered in May 2021 release of CPRD Aurum. Controls were matched for year of birth, sex and GP and if their start of record was no later than 18 months after that of the matched DS participant. The final cohort comprised 8854 DS patients (4639 males) who were matched to 34,724 controls (18,276 males).

### Main Measures

We identified COVID-19 diagnosis using Read codes for confirmed or suspected COVID-19 from patients’ clinical records as reported by CPRD.^12^ Suspected diagnoses were included because of the limited availability of testing during the first wave of the pandemic. We excluded COVID-19 diagnoses dated before the 29th of January 2020. We evaluated comorbidities from patients’ clinical records using Read codes from the OpenSAFELY study^19^ and code lists created for DS-specific conditions. These Read code lists were created and then verified by a second reviewer for consensus. If consensus was not met, an external third reviewer with expertise in the area was consulted for their input.

Read codes for the following diagnoses were created: congenital heart disease, dementia, hypothyroidism, other chronic inflammatory conditions/other autoimmune conditions (including rheumatoid arthritis, alopecia and psoriasis), chronic respiratory disease excluding asthma (including chronic obstructive pulmonary disease, pneumonias and cystic fibrosis), asthma, cerebrovascular accident, ischaemic heart disease/peripheral vascular disease, hypertension, other cardiovascular diseases (including conduction and rhythm disorders, cardiomyopathies and valvular disease), chronic liver disease, chronic kidney disease, obesity, diabetes (both type 1 and type 2), epilepsy, anxiety disorders, depression, sleep disorders, cancer (including solid tumours, skin cancers and metastatic disease) and haematological malignancy, as well as for smoking status and living situation (living in residential or care settings).

### Statistical Analysis

We fitted conditional logistic regression models to estimate associations between DS and COVID-19 diagnosis, compared to general population controls, reporting odds ratios (OR) with 95% confidence intervals (95% CI). We adjusted for DS-specific and non-specific comorbidities in the models. Interaction analyses were conducted between DS and clinically relevant comorbidities, in this instance, chronic respiratory disease (except asthma), dementia and asthma, to examine how these might impact susceptibility to COVID-19 diagnosis. We fitted conditional logistic regression models to explore the associations between multimorbidity and susceptibility to COVID-19 diagnosis. Multimorbidity was fitted as a factor ranging from 1 to 7 and 8+ comorbidities. Finally, an exploratory logistic regression model was used to examine the potential impact of living situation on susceptibility to COVID-19 diagnosis in a sub-sample of patients with living situation data. In this model, we adjusted for age group, gender and DS-specific and non-specific comorbidities.

## RESULTS

We analysed data for the period January 2020 to May 2021 and the cohort comprised 43,578 patients.

### Pattern of Infection Rates During the Pandemic

**Figure 1:**
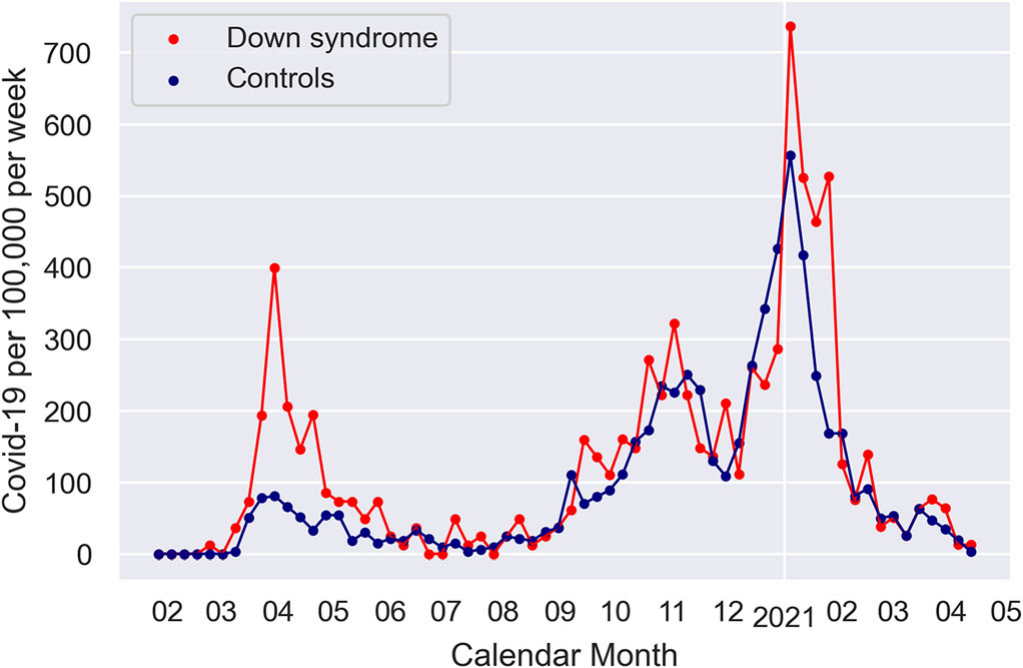
COVID-19 diagnosis per 100,000 per week for DS and controls.

As Fig. 1 shows, the excess of COVID-19 diagnoses in DS individuals was more prominent during the peak periods of the pandemic in April/May 2020 and November/December 2020. The excess rates in people with DS in the second wave of the pandemic occurred despite enhanced awareness of protective measures such as wearing masks and limiting social contacts, suggesting that either these measures were not sufficient to protect this high-risk group or otherwise were not consistently applied.

Table 1 shows the demographic characteristics and comorbidities of DS cases and general population controls according to COVID-19 diagnosis. As expected, a higher proportion of people with DS were diagnosed with COVID-19 compared to controls (7.4% vs 5.6%, *p* ≤ 0.001).

**Table 1:** Demographic Information and Prevalence of Comorbidities of the DS Group and Controls by COVID-19 Diagnosis (%)

		DS group (*n* = 8854)			Controls (*n* = 34,724)		
		COVID-19 diagnosis	No COVID-19 diagnosis	*p*	COVID-19 diagnosis	No COVID-19 diagnosis	*p*
*n*		651 (7.4)	8203 (92.6)		1936 (5.6)	32,788 (94.4)	
Mean age (SD)		38.26 (20.07)	28.64 (19.16)	< 0.001	32.34 (17.56)	28.90 (19.52)	< 0.001
Age group	0–9 years	68 (10.4)	1699 (20.7)	< 0.001	227 (11.7)	6838 (20.9)	< 0.001
	10–19 years	74 (11.4)	1540 (18.8)		273 (14.1)	6160 (18.8)	
	20–29 years	92 (14.1)	1249 (15.2)		417 (21.5)	4868 (14.8)	
	30–39 years	89 (13.7)	1144 (13.9)		327 (16.9)	4459 (13.6)	
	40–49 years	71 (10.9)	969 (11.8)		271 (14.0)	3623 (11.0)	
	50–59 years	155 (23.8)	1130 (13.8)		317 (16.4)	4691 (14.3)	
	60–69 years	90 (13.8)	398 (4.9)		93 (4.8)	1825 (5.6)	
	70–79 years	12 (1.8)	74 (0.9)		11 (0.6)	324 (1.0)	
Gender	Male	348 (53.5)	4291 (52.3)	0.596	918 (47.4)	17,358 (52.9)	< 0.001
	Female	303 (46.5)	3912 (47.7)		1018 (52.6)	15,430 (47.1)	
Congenital heart disease		167 (25.7)	2968 (36.2)	< 0.001	28 (1.4)	277 (0.8)	0.011
Anxiety disorders		67 (10.3)	615 (7.5)	0.014	375 (19.4)	4484 (13.7)	< 0.001
Other chronic inflammatory conditions/other autoimmune conditions		395 (60.7)	4182 (51.0)	< 0.001	786 (40.6)	12,033 (36.7)	0.001
Cerebrovascular accident		18 (2.8)	133 (1.6)	0.039	19 (1.0)	267 (0.8)	0.436
Dementia		117 (18.0)	501 (6.1)	< 0.001	1 (0.1)	17 (0.1)	1
Depression		78 (12.0)	560 (6.8)	< 0.001	321 (16.6)	3962 (12.1)	< 0.001
Diabetes (both type 1 and type 2)		90 (13.8)	726 (8.9)	< 0.001	128 (6.6)	1412 (4.3)	< 0.001
Epilepsy		169 (26.0)	1150 (14.0)	< 0.001	78 (4.0)	1051 (3.2)	0.055
Ischaemic heart disease/peripheral vascular disease		24 (3.7)	215 (2.6)	0.13	48 (2.5)	722 (2.2)	0.426
Hypertension		18 (2.8)	162 (2.0)	0.191	172 (8.9)	1993 (6.1)	< 0.001
Hypothyroidism		253 (38.9)	2559 (31.2)	< 0.001	66 (3.4)	707 (2.2)	0.001
Obesity		96 (14.7)	938 (11.4)	0.013	151 (7.8)	1555 (4.7)	< 0.001
Other cardiovascular disease		86 (13.2)	925 (11.3)	0.153	68 (3.5)	793 (2.4)	0.003
Sleep disorders		146 (22.4)	1545 (18.8)	0.029	176 (9.1)	2355 (7.2)	0.002
Ever smoked		64 (9.8)	721 (8.8)	0.352	684 (35.3)	9464 (28.9)	< 0.001
Asthma		104 (16.0)	1084 (13.2)	0.049	412 (21.3)	5089 (15.5)	< 0.001
Cancer		13 (2.0)	146 (1.8)	0.645	60 (3.1)	781 (2.4)	0.057
Haematological malignancy		11 (1.7)	160 (2.0)	0.767	8 (0.4)	121 (0.4)	0.699
Chronic liver disease		8 (1.2)	20 (0.2)	0.001	7 (0.4)	85 (0.3)	0.36
Chronic kidney disease		60 (9.2)	383 (4.7)	< 0.001	26 (1.3)	327 (1.0)	0.16
Chronic respiratory disease excluding asthma		181 (27.8)	1114 (13.6)	< 0.001	83 (4.3)	1052 (3.2)	0.012

In the DS group, people diagnosed with COVID-19 had higher rates of anxiety disorders, other chronic inflammatory conditions/other autoimmune conditions, cerebrovascular accidents, dementia, depression, diabetes (both type 1 and type 2), epilepsy, hypothyroidism, obesity, sleep disorders, asthma, chronic liver disease, chronic kidney disease and chronic respiratory disease compared to people with DS without a COVID-19 diagnosis. For controls, those with a COVID-19 diagnosis had higher rates of congenital heart disease, anxiety disorders, other chronic inflammatory conditions/other autoimmune conditions, depression, diabetes (both type 1 and type 2), hypertension, hypothyroidism, obesity, other cardiovascular diseases, sleep disorders, smoking, asthma and chronic respiratory disease compared to those without COVID-19.

Unadjusted for comorbidities listed in Table 1, we found that DS cases had an increased likelihood of receiving a COVID-19 diagnosis compared to controls (Table 2). In a model adjusted for comorbidities, COVID-19 diagnosis was most strongly associated with dementia (OR = 2.75, 95% CI 2.03–3.72), chronic respiratory disease excluding asthma, epilepsy, hypertension, asthma, depression, anxiety disorders and other chronic inflammatory conditions/other autoimmune conditions explaining increased susceptibility of COVID-19 diagnosis. DS was not independently associated with COVID-19 after adjusting for comorbidities.

**Table 2:** Results of the Unadjusted and Adjusted Conditional Regression Models Examining the Associations Between DS and Susceptibility to COVID-19 Diagnosis

Variable	OR	95% confidence interval		*p*
		LL	UL	
Model 1				
DS diagnosis	1.35	1.23	1.48	< 0.0001
Model 2				
DS diagnosis	1.12	0.97	1.29	0.121
Dementia	2.75	2.03	3.72	< 0.0001
Chronic respiratory disease excluding asthma	1.48	1.24	1.76	< 0.0001
Epilepsy	1.28	1.07	1.53	0.008
Hypertension	1.24	1.01	1.52	0.040
Asthma	1.24	1.10	1.40	< 0.001
Cancer	1.21	0.90	1.62	0.209
Depression	1.18	1.02	1.37	0.024
Anxiety disorders	1.17	1.02	1.34	0.026
Chronic liver disease	1.39	0.71	2.73	0.333
Other cardiovascular disease	1.12	0.90	1.38	0.304
Other chronic inflammatory conditions/other autoimmune conditions	1.11	1.01	1.23	0.029
Diabetes (both type 1 and type 2)	1.11	0.92	1.34	0.288
Cerebrovascular accident	1.06	0.70	1.61	0.793
Hypothyroidism	1.05	0.89	1.25	0.549
Obesity	1.05	0.88	1.25	0.605
Chronic kidney disease	1.03	0.77	1.38	0.846
Sleep disorders	1.01	0.87	1.17	0.924
Ever smoked	0.94	0.84	1.06	0.303
Ischaemic heart disease/peripheral vascular disease	0.90	0.67	1.20	0.464
Congenital heart disease	0.84	0.68	1.02	0.084
Haematological malignancy	0.61	0.35	1.06	0.080

### Interactions Between Comorbidities and Susceptibility to COVID-19 Diagnosis

Comorbidities from our previous models were chosen based on their clinical relevance; these included chronic respiratory disease, asthma and dementia. We found evidence that the association of DS with COVID-19 varied by chronic respiratory disease status. People with DS who had a chronic respiratory disease were more susceptible to being diagnosed with COVID-19, OR = 1.71 (95% CI 1.20–2.43), *p* = 0.003. Interactions with asthma (*p* = 0.28) and dementia (*p* = 0.36) diagnosis did not improve goodness of fit.

### Risk Associated with Multimorbidity

As Table 3 shows, multimorbidity was associated with increased susceptibility to a COVID-19 diagnosis compared to having no comorbidities. Adjusting for multimorbidity, people with DS were still more likely to receive a COVID-19 diagnosis compared to controls.

**Table 3:** Results of the Unadjusted and Adjusted Conditional Logistic Regression Models Examining Associations Between Multimorbidity and Susceptibility to COVID-19 Diagnosis

Variable	OR	95% confidence interval		*p*
		LL	UL	
Model 1				
DS diagnosis	1.35	1.23	1.48	< 0.0001
Model 2				
DS diagnosis	1.11	1.00	1.23	0.045
Number of comorbidities				
0	Ref.			
1	1.18	1.04	1.34	0.010
2	1.42	1.23	1.64	< 0.0001
3	1.47	1.24	1.74	< 0.0001
4	1.79	1.47	2.18	< 0.0001
5	1.89	1.46	2.45	< 0.0001
6	2.31	1.66	3.21	< 0.0001
7	2.39	1.55	3.69	< 0.0001
8+	3.49	2.03	6.01	< 0.0001

### Impact of Living Situation on Susceptibility to COVID-19

To examine the effect of living situation on the DS and chronic respiratory disease diagnosis interaction, we fitted a standard logistic regression on a sub-sample of 4528 patients (10.4% of total sample) who had data on their living situation. Living situation data was coded as per the OpenSAFELY study^20^ comparing those living in residential or care settings with those in other living situations (living with relatives etc.).

When adjusting for the impact of living in residential or care settings, the DS and chronic respiratory disease diagnosis interaction was not significant, OR = 1.83 (95% CI 0.86–4.28), *p* = 0.14. In this model, age groups 50–59 (OR = 4.32; 95% CI 1.30–26.80; *p* = 0.04); 60–59 (OR = 4.70; 95% CI 1.38–29.49; *p* = 0.04) and 70–79 (OR = 5.02; 95% CI 1.29–33.37; *p* = 0.04), epilepsy (OR = 1.46; 95% CI 1.14–1.84; *p* = 0.002) and living in residential or care settings (OR = 1.60; 95% CI 1.27–2.01; *p* < 0.0001) were associated with increased likelihood of a COVID-19 diagnosis.

## DISCUSSION

This analysis of large-scale population-level UK primary care data identified that people with DS had a significantly increased likelihood of receiving a COVID-19 diagnosis compared to controls (OR = 1.35; 95% CI 1.23–1.48), and this was not entirely explained by multimorbidity. We found that susceptibility to COVID-19 diagnosis across the cohort was associated with specific comorbidities including anxiety disorders, other chronic inflammatory conditions/other autoimmune conditions, dementia, depression, epilepsy, hypertension, asthma and chronic respiratory disease excluding asthma. In particular, people with DS and a chronic respiratory disease diagnosis were significantly more susceptible to COVID-19 (OR = 1.71; 95% CI 1.20–2.43). However, this did not remain significant when controlling for exposure through living situation in a smaller sub-sample of patients with data available on living situation.

Our findings of an interaction between chronic respiratory conditions in DS and risk of COVID-19 diagnosis may indicate a common mechanism for increased risk for respiratory conditions in general in DS individuals, or alternatively that existing respiratory vulnerability (excluding asthma) may be associated with increased susceptibility to infection with SARS-CoV-2. DS is associated with certain anatomical differences, facial and airway tract features that may increase risk for respiratory infections, such as macroglossia, a narrow nasopharynx and a shortened palate, further complicated by generalised hypotonia.^21^ These may be associated with increased risk for ear, nose and throat infections^22^ and obstructive sleep apnoea.^23^ Furthermore, a higher prevalence of congenital cardiac anomalies and pulmonary hypertension^24^ may further increase the risk for respiratory infections in people with DS.

Ultimately, respiratory infections are a significant source of increased morbidity and mortality in children and adults with DS.^25^ Approximately 40% of children and young adults (aged < 30 years) with DS experience at least one episode of pneumonia.^26^ Pneumonia was associated with higher risk of inpatient mortality when adjusting for age, gender and ethnicity for adults with DS compared to controls using data from the UK.^27^

Type 1 interferons (IFNs) help to regulate the activity of the immune system. Trisomy 21 is characterised by enhanced IFN-1 signalling due to overexpression of IFN genes on chromosome 21 (IFNAR1, IFNAR2 and IFNGR2). The effect on respiratory infections are difficult to predict, though may have both positive and negative effects by enhancing anti-viral response and overactivation of the immune system with risk for a cytokine storm.^7^ Individuals with DS may also have reduced lymphocyte numbers.^28^ Immune dysregulation has been proposed to be a syndromic feature,^29^ and the cell-mediated immunodeficiency combined with impaired antibody response to pathogens may explain the increased risk for and mortality associated with pneumonia and other respiratory diseases in children with DS.

Given the biological risk associated with increased viral entry, individuals with DS may also have a particular predisposition for viral respiratory infections in general, rather than specifically for SARS-CoV-2. *TMPRSS2* belongs to the type II transmembrane serine protease family and is commonly expressed in epithelial tissues such as those lining the upper airways, bronchi and lung.^30,31^ Influenza viruses and other coronaviruses all critically depend on *TMPRSS2* for viral activation and cellular infection.^31,32^ Viruses such as *influenza virus, respiratory syncytial virus* (*RSV*) and *parainfluenza virus* likely account for most of the respiratory infections in DS,^22,33^ and those with congenital cardiac conditions or pulmonary hypertension may be particularly vulnerable.

Obesity is a common comorbidity in DS, and associated with increased disease severity when diagnosed with COVID-19 in the general population, probably through its association with decreased lung reserve volume and capacity, as well as reduced pulmonary function in bed-bound patients. Obesity is also associated with higher levels of inflammatory cytokines, which may contribute to higher mortality rates in obese individuals with COVID-19.^34^ In the present study, obesity was not associated with increased susceptibility to COVID-19 when adjusting for other comorbidities. This suggests that the increased risk associated with obesity may be related to complications and treatment outcomes rather than susceptibility to infection. Additionally, congenital heart disease may be associated with higher risk for hospitalisation in those with COVID-19 infection,^10^ but although 35% of DS participants had a diagnosis of congenital heart conditions in our study, it was not associated with increased susceptibility to infection.

Our data and other research suggest that individuals with DS are particularly at risk for COVID-19 and other respiratory infections. Our data further emphasises the need to prioritise individuals with DS for COVID-19 vaccination. They should also be prioritised for surveillance of respiratory infections during high-risk periods,^35^ and for implementing additional safety measures during pandemics. This may include preventing and rapidly containing transmission in long-term care facilities, providing support to caregivers on close monitoring and safe care, and identification of symptoms that may indicate the need for assessment for admission to hospital. Active medical management to ensure access to oxygen therapy and drugs such as steroids and antibiotics when needed could prevent complications and hospital admissions. There may also be a rationale for offering pneumococcal vaccine in addition to flu vaccine to children and adults with DS. Other preventative measures include management of comorbidities such as obesity and treatment of sleep apnoea.^36^ Future studies on the contribution of *TMPRSS2* triplication and developing therapies that may be particularly relevant to DS are needed.

This is the largest study to date of COVID-19 in individuals with DS, including nearly 9000 DS individuals, of whom 651 were diagnosed with COVID-19. We included data from the whole of 2020 and part of 2021, thus covering the initial and subsequent waves of the pandemic in the UK. Nevertheless, differential access to testing might have been a potential source of bias due to limited access to tests during the first wave of the pandemic.^37^ Whilst the probability of an infected person receiving a test might have been variable over time, it is unlikely to account for differences in rates between individuals with DS and general population controls since it affected all patients and GPs across the UK. It is possible that those with symptomatic infections were more likely to be tested and recorded with an infection by their primary care physicians than asymptomatic cases. Whether people with DS are less likely to experience asymptomatic infections is currently unknown. Our exploratory sub-analysis examining the impact of living situation on COVID-19 diagnosis found that living in residential or care settings may increase the likelihood of COVID-19 diagnosis. This finding is consistent with the excess mortality and infection rates reported in the UK^38^ and internationally^39^ for those living in residential or care settings. Whilst living situation was an important risk factor for COVID-19 diagnosis, this analysis was only conducted in 10% of the total sample with living situation data available and only 2.3% (800/34,724) of controls had data on this variable. Therefore, the interaction between living situation and chronic respiratory disease in those with DS could not be fully examined, and future studies with better recording of living situation data are needed. Further, it is not yet clear how multimorbidity and health-seeking behaviours may have influenced COVID-19 diagnosis. Multimorbidity is associated with increased primary care use^40^ which may have increased the likelihood of patients consulting with their GP for COVID-19 symptoms. Therefore, the relationship between COVID-19 diagnosis and multimorbidity warrants further investigation.

Individuals with DS have both increased susceptibility to COVID-19 infection and are more likely to experience severe outcomes. Susceptibility to COVID-19 is increased during peaks of the pandemic and associated with health comorbidities in individuals with DS, particularly chronic respiratory conditions. Although lockdowns and the associated measures to wear masks and social distancing appeared to have an effect in reducing the initial wave of the pandemic, it did not seem to prevent excess infections in DS individuals during the 2^nd^ wave in the autumn winter of 2020, suggesting that these measures were not fully utilised for this group, or else that additional safeguards may be required. This has important public health implications to manage risk during the ongoing SARS-CoV-2 pandemic, as well as to manage the risk associated with respiratory infections in people with DS. Individuals with DS who have underlying lung conditions should take extra care to safeguard themselves during viral pandemics, and our findings suggest a strong rationale for them to be prioritised for vaccination with COVID-19, influenza and pneumococcal vaccines.

## Data Availability

Data are available upon reasonable request. Requests for access to data from the study should be addressed to martin.gulliford@kcl.ac.uk. All proposals requesting data access will need to specify planned uses with approval of the study team and CPRD before data release.
